# Psychometric validation of the severity of chronic cough diary, leicester cough questionnaire, and a cough severity visual analogue scale in patients with refractory chronic cough

**DOI:** 10.1186/s41687-025-00888-z

**Published:** 2025-06-11

**Authors:** Andrew Trigg, Nathan Clarke, Christoph Gerlinger, Ulrike Krahn, Adam Gater, Claudia Haberland

**Affiliations:** 1 Bayer plc, Reading, UK; 2 Adelphi Values, Macclesfield, UK; 3 Bayer AG, Berlin, Germany; 4 Department of Gynecology, Obstetrics and Reproductive Medicine, University Medical School of Saarland, Homburg/Saar, Germany; 5 Bayer AG, Wuppertal, Germany

**Keywords:** Refractory chronic cough, Severity of chronic cough diary, Leicester cough questionnaire, Cough severity VAS, Patient-reported outcome, Psychometric validation, Reliability, Validity, Meaningful change

## Abstract

**Background:**

Refractory chronic cough (RCC) is commonly reported in primary care and associated with significant morbidity. Patient-reported outcome (PRO) measures are important for evaluating the efficacy of antitussive medications for RCC in clinical trials from the patient-perspective. Psychometric properties of Severity of Chronic Cough Diary (SCCD) Cough Severity and Cough Frequency, Leicester Cough Questionnaire (LCQ) Total and Physical Domain and Cough Severity Visual Analogue Scale (VAS) scores using data from a 12-week Phase 2b trial evaluating the efficacy of eliapixant in patients with RCC (NCT04562155) are reported.

**Results:**

Quality of completion for the SCCD, LCQ and Cough Severity VAS across the study was high, no ceiling or floor effects were observed at baseline. Internal consistency for LCQ Total and Physical domain scores was also high (Cronbach’s alpha = 0.939 and 0.806, respectively). SCCD Cough Frequency and Cough Severity, LCQ Total and Physical domain, and Cough Severity VAS scores demonstrated strong test-retest reliability (Intraclass correlation coefficient ≥ 0.848) among participants defined as stable between Week 3 and Week 4 according to Patient Global Impression of Severity (PGI-S) ratings and Awake Cough Count readings. Construct validity was supported by known-groups comparisons, with large differences (effect sizes 1.99–4.16) observed between groups categorized according to PGI-S ratings and objective Awake Cough Counts. Ability to detect improvement was supported by large effect sizes (≥0.8) observed for mean changes in SCCD, LCQ and Cough Severity VAS scores from baseline to Week 12 among participants classified as ‘improved’ according to PGI-S/PGI-C ratings and Awake Cough Counts. Triangulated thresholds (score range) for meaningful within-patient improvement based on anchor-based assessments were -0.82 for SCCD Cough Frequency (0–4), -0.69 for SCCD Cough Severity (0–4), 2.36 for the LCQ Total (3–21), 0.77 for the LCQ Physical (1–7) and -17.73 for the Cough Severity VAS (0–100) scores.

**Conclusion:**

Findings support the reliability, validity and responsiveness of the newly developed SCCD Cough Frequency and Severity items as fit-for-purpose PRO measures of cough frequency or severity for use in drug development programs within RCC. The LCQ Total, LCQ Physical Domain and Cough Severity VAS also exhibit acceptable measurement properties for use in this population.

**Supplementary Information:**

The online version contains supplementary material available at 10.1186/s41687-025-00888-z.

## Background

Refractory chronic cough (RCC) refers to a pervasive cough (lasting longer than 8 weeks), with unexplained underlying aetiology being unresponsive to conventional treatment [1]. Affecting approximately 10% of adults globally [2], morbidity associated with RCC is considerable with significant detrimental impact of the symptoms on patients’ health-related quality of life (HRQoL) [3–6].

Changes in cough frequency, measured by objective monitoring devices, typically serve to assess primary efficacy endpoints in clinical trials for RCC therapies [7–9]. However, to determine the clinical benefit of medical interventions on how patients ‘feel, function and survive’ and to provide a holistic view of the patient experience of RCC, it is recommended that objective methods be supplemented via patient-reported outcome (PRO) measures [10]. This is consistent with guidance from regulatory agencies e.g. the US Food and Drug Administration (FDA) and European Medicines Agency (EMA) which emphasize the importance of patient perspectives in treatment evaluation [11–13].

Prior research highlights cough frequency, severity and associated impacts on patients’ daily lives and overall HRQoL as meaningful and important concepts to assess when evaluating outcomes of treatment for RCC [14–16]. Indeed, it has been acknowledged that any reduction in the symptoms of cough needs to be strongly correlated with improvements in HRQoL to be considered clinically meaningful and patient-relevant [17]. The Leicester Cough Questionnaire (LCQ) is a cough-specific measure of HRQoL frequently used in research studies [18, 19]. Prior research has supported the LCQ as a reliable, valid and responsive measure of the impact of RCC on patients’ lives [18, 20]. However, the LCQ does not provide a direct assessment of cough frequency and severity which is important for determining the effectiveness of interventions for treating RCC [21].

Historically, cough severity in RCC clinical trials and clinical research has been assessed via a single-item Cough Severity Visual Analogue Scale (VAS). The Cough Severity VAS is not a ‘standardised’ assessment but it captures patients’ self-assessment of cough severity while often ranging from 0 mm (no cough) to 100 mm (worst cough) [22]. The Cough Severity VAS has demonstrated evidence of reliability, validity and responsiveness among RCC populations [23]. However, VAS measures are subject to inherent limitations which may limit their utility for evaluating treatment effect and supporting clinical trial endpoints. Most notably, ceiling effects may be observed for VAS measures [24], and there exist concerns over participants ability to make fine distinctions along a 100 mm scale [23]. Further, as a single item, the Cough Severity VAS might not appropriately capture all aspects of cough severity, with evidence suggesting that cough severity is a function of a variety of different factors including frequency, intensity, disruption to sleep and daily activities.

Recent clinical studies investigating antitussive medications in RCC have made use of proprietary diaries to assess the patient experience of cough in a manner that conforms to regulatory expectations and best practices for PRO measures. This includes the Cough Severity Diary (CSD) [25, 26] used in clinical trials to evaluate the efficacy of gefapixant [27, 28]. Due to restrictions on the use of the CSD for commercial research purposes, Bayer developed the Severity of Chronic Cough Diary (SCCD) for use in trials to evaluate the efficacy and safety of eliapixant [29] in accordance with scientific guidance and best practice [11–13, 30, 31]. Prior qualitative research with RCC patients and continuous expert involvement throughout development supports the content validity of the SCCD in RCC [32]. However, for any PRO measures intended to assess endpoints in clinical trials, evidence of psychometric validity and measurement properties (including definitions of meaningful within patient-change [MWPC]) within the designated context of use [11–13, 33] is mandatory.

This article summarizes findings from evaluation of the psychometric properties of selected scores derived from the SCCD, LCQ and Cough Severity VAS using data from a Phase 2b randomized control trial among patients with RCC.

## Methods

### Study design and participants

PAGANINI (ClinicalTrials.gov NCT04562155) was a randomized, double-blind, parallel-group, placebo-controlled, dose-finding efficacy and safety study comparing twice-daily oral administration of three different doses of eliapixant to placebo in patients with RCC over a period of 12 weeks [29]. Adults aged ≥ 18 years with RCC lasting ≥ 12 months, with persistent cough for ≥ 8 weeks before screening, and with cough severity ≥ 40 mm measured on a 100 mm VAS at screening, were enrolled by study investigators. Participants with a range of range of RCC-related medical conditions were excluded (e.g., those with history of smoking history or exposure to inhalational toxic fumes within the last 12 months; presence of lung disease that could be responsible for cough; recent respiratory tract infection). The study protocol and statistical analysis plan are available on ClinicalTrials.gov.

### Outcome assessments

**Fig. 1:**
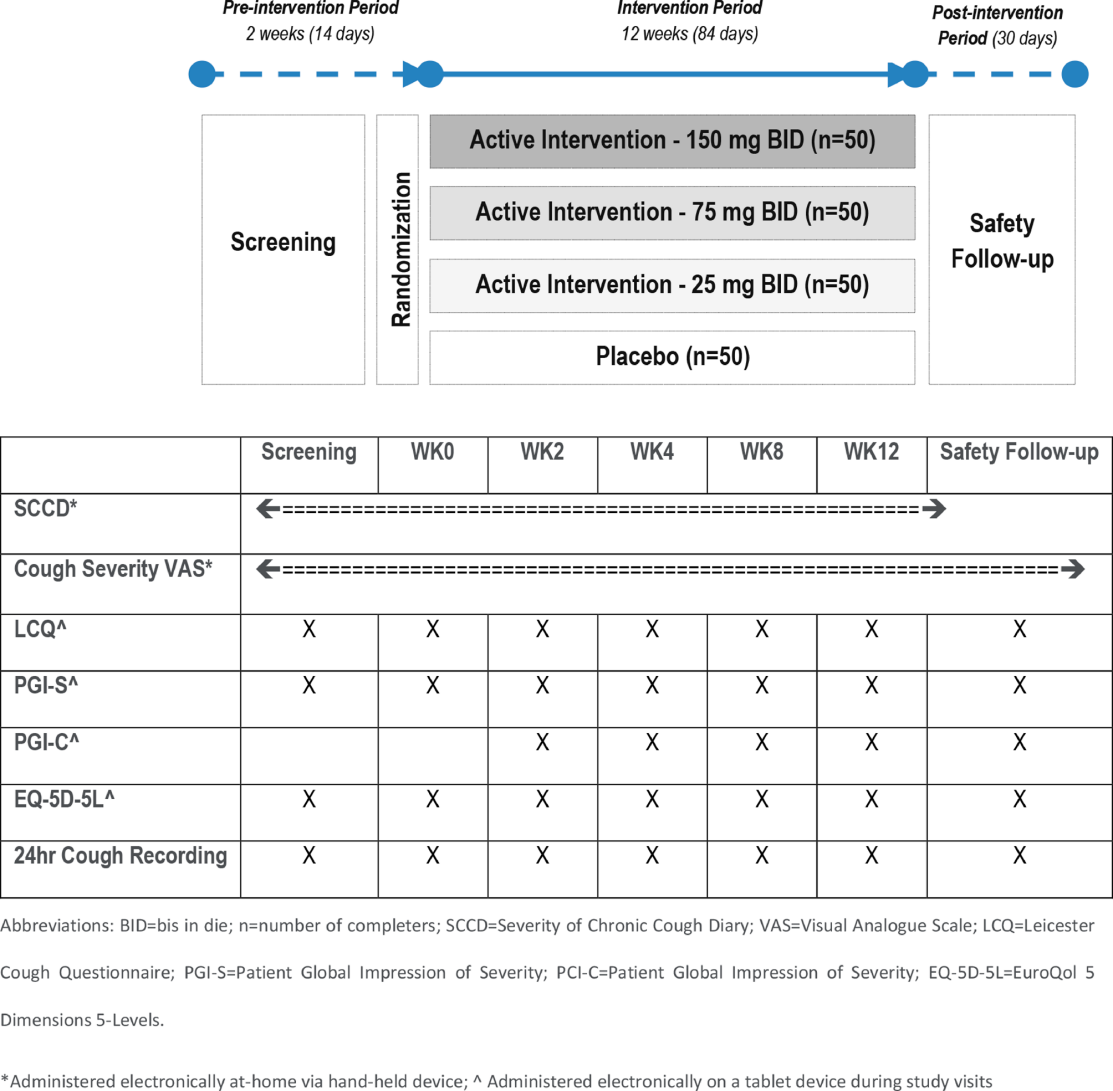
Study design and schedule of COA assessments

Participants completed the SCCD and Cough Severity VAS every day throughout the two-week screening period and 12-week intervention period. At every visit from screening to Week (WK) 12, participants also completed the LCQ, PGI-S, PGI-C (except for screening and WK0) and EQ-5D-5L. An ambulatory recording device (VitaloJAK, Vitalograph, Ireland) [34] was used to perform 24 hr cough count monitoring at each of these visits (Fig. 1). The change from baseline in 24-hour cough count after 12 weeks of intervention (measured by Vitalojak) was the primary endpoint in the PAGANINI study, guiding the sample size calculation for the trial. All PROs assessed secondary or exploratory endpoints.

The following PRO measures are analysed as part of this study:

- The SCCD (v1.0) is a 14-item PRO measure asking respondents to assess experiences with their cough during the past 24 hours [32]. Five items directly assess respondents experience of cough with respect to frequency, severity, coughing fits, urge to cough, and control of cough. Six items assess other symptoms associated with cough (including pain, physical discomfort, breathlessness, incontinence due to cough, difficulty falling asleep and disturbed sleep). The remaining three items assess limitations to usual activities. Items utilise categorical verbal rating scales (VRS), whereby lower scores equate to better health (except for Item 4 ‘In the past 24 hours, how often were you able to control your cough?’). In the PAGANINI study SCCD items were analysed individually. As concepts of cough frequency and severity were highlighted as being of primary importance to RCC patients [16] and typically used to assess key efficacy endpoints in clinical studies, analyses reported herein focus specifically on SCCD Item 1 (Cough Frequency) and Item 5 (Cough Severity) scores.
- The LCQ is a 19-item PRO measure that asks about the impact of chronic cough on various aspects of participants’ lives using a recall period of two weeks. Three domain scores can be calculated as the average of the item responses in the respective domain (Physical Domain – 8 items; Psychological Domain – 7 items; Social Domain – 4 items). All LCQ single item and domain scores are ranging from 1 (all of the time) to 7 (none of the time) with higher scores indicating better HRQoL. Individual domain scores can be summed to provide a LCQ Total score ranging from 3 to 21. Analyses in this manuscript are focused on the LCQ Total score and LCQ Physical domain score. Prior research has indicated a 1.3 to 2.3 point increase for the LCQ Total score and ≥0.8 for the LCQ physical domain score to represent a clinically significant improvement [20, 35].
- The Cough Severity VAS is a single item asking the study participant to indicate the severity of their cough during the past 24 hours using a vertically oriented line ordered from 0 (No cough) to 100 (Extremely severe cough). Prior research has indicated that a cough severity VAS reduction of ≥ 20 mm reflects a minimally important change and ≥ 30 mm reflects a larger threshold for those who are much improved [23].

In addition, the following outcome assessments were used as anchors or to support the analyses:

- The Patient Global Impression of Severity (PGI-S) is a single item assessing severity of cough (no recall period) using a 6-point VRS: a score of 0 = “No cough”, 1 = “Very mild”, 2 = “Mild”, 3 = “Moderate”, 4 = “Severe”, 5 = “Very severe.
- The Patient Global Impression of Severity (PGI-C) is a single item assessing change in the severity of cough since starting study medication using a 7-point VRS: a score of 0 = “Very much better”, 1 = “Much better”, 2 = “A little better”, 3 = “No change”, 4 = “A little worse”, 5 = “Much worse” and 6 = “Very much worse”.
- The EQ-5D-5 L is a self-administered generic and widely used measure of health status with well-documented reliability, validity and responsiveness in the general population as well as in various diseases [36].
- Objective 24-hour cough monitoring was performed with the VitaloJAK cough recorder (VitaloJAK, Vitalograph). This is a non-invasive, belt-worn, battery operated, and custom-built validated recording device and microphone used to acquire, record and store ambulatory cough sounds from participants for 24 hours at screening and week (WK)s 0, 2, 4, 8 and 12. Dual sensors overcame recording problems from muffling caused by PPE masks and cough suppression. Also silences and background noise were removed by using validated, custom-written software, and cough sounds were counted by using an audio editing package (Vitalograph). The number of coughs is expressed as coughs per hour and added up to give a cough count for a 24-hour period and during periods of awakening (Awake Cough Count). Prior research has supported the reliability and validity of the Vitalojak for objective cough monitoring, with a ≥ 30% reduction defined as a meaningful change threshold to define treatment response in RCC clinical trials [37, 38].

### Statistical analysis

Analyses were conducted on data for all participants who had at least one item completed on the SCCD at any timepoint. Descriptive statistics [mean, standard deviation (SD) for continuous data, frequencies for categorical data] were calculated for sample clinical and demographic characteristics at baseline.

The measurement properties of SCCD Item 1 (Cough Frequency), SCCD Item 5 (Cough Severity), LCQ Total, LCQ Physical domain and Cough Severity VAS scores were evaluated in accordance with regulatory standards and expectations for use of PRO measures in drug development [11–13, 33]. No imputations were performed for any PRO scores to account for missing data. All analyses were conducted using SAS version 9.4 unless otherwise specified. In a psychometric validation study the emphasis is on evaluating the magnitude of relationships between variables and the overall pattern of results. Where significance testing was conducted (using a threshold of p < 0.05) it is considered exploratory and no adjustments for multiplicity of tests were used.

Analyses completed for each PRO measure are summarized in Table 1. Further details of specific analyses are provided as follows:

- **Quality of completion and descriptive statistics:** The frequency and percentage of participants with form-level missing data for the SCCD, LCQ and Cough Severity VAS at baseline (BL), WK4 and WK12 (SCCD and Cough Severity VAS scores based on 7 days up to and including the respective study visit) was reported. The proportion of participants with the highest and lowest possible score(s) for the SCCD, LCQ and Cough Severity VAS at BL was also reported. Floor and ceiling effects were defined as instances where > 15% of participants had the most or least severe possible score respectively [39].
- **Reliability:** Internal consistency of multi-item scores (i.e., LCQ Total and LCQ Physical domain scores) was investigated by calculating Cronbach’s alpha coefficient for each score at WK4, with alpha coefficients ≥ 0.70 considered acceptable [40]. Test-retest reliability between WK2 and WK4 was investigated for SCCD Item 1 (Cough Frequency), SCCD Item 5 (Cough Severity) and Cough Severity VAS weekly average scores, as well as LCQ Total and LCQ Physical domain scores. Test-retest reliability was investigated by calculating the intraclass correlation coefficient (ICC) for ‘stable’ subjects (comparing data from WKs 2 and 4, with change defined in two ways: 1) change of 0 in the PGI-S; 2) change < 30% in Awake Cough Count (as measured by the VitaloJAK cough recorder). For weekly SCCD and Cough Severity VAS scores an ICC based on a multiple measurement, absolute agreement, two-way mixed effects model (using the terminology of Koo and Li) was used [41]. ICCs for LCQ scores were based on a single measurement, absolute agreement, two-way mixed effects model [41, 42]. ICC values less than 0.50 were considered indicative of poor reliability, values between 0.50 and 0.75 indicative of moderate reliability, values between 0.75 and 0.90 indicative of good reliability, and values greater than 0.90 indicated excellent reliability [41].
- **Construct validity:** Relationships between PRO scores were evaluated through multi-trait analysis (LCQ only as it is a multi-item score) and assessment of convergent/divergent validity (SCCD only to avoid circular comparisons for the LCQ and Cough Severity VAS), known-groups comparisons and evaluation of ability to detect change.
  - Multi-trait analysis was performed to evaluate the appropriateness of the domain structure and associated scoring algorithms for the LCQ at WK4 by evaluating item-scale polyserial correlations applying the following criteria for acceptability: The correlation coefficient between each item and its own corrected domain (i.e., excluding the item being investigated from the domain score) should be at least 0.40 and each item should have a higher correlation coefficient with its own corrected domain than with any other domain [43, 44].
  - Convergent validity correlations between SCCD Item 1 (Cough Frequency) and SCCD Item 5 (Cough Severity) weekly scores and other concurrent measures (e.g., LCQ, Cough Severity VAS, PGI-S, EQ-5D-5 L, objective awake/night cough counts) were examined at WK4. Relationships where the resulting coefficient was < 0.3 were classified as weak; ≥ 0.3 to < 0.5 were classified as moderate; ≥ 0.5 to < 0.7 were classified as high; and ≥ 0.7 classified as very large [45, 46]. Spearman’s (for investigation of relationships between continuous variables) or polyserial correlations (for investigation of relationships between continuous and ordinal variables) were used. Moderate to high correlations (≥ 0.3) were hypothesized between SCCD Item 1 (Cough Frequency) and SCCD Item 5 (Cough Severity) scores with scores from disease-specific measures (LCQ, Cough Severity VAS, PGI-S) and Awake Cough Counts. By contrast, weak correlations were (<0.3) hypothesized with generic measures (EQ-5D scores) and Night Cough Counts
  - Known groups comparisons exploring the ability of SCCD, LCQ and Cough Severity VAS scores to differentiate between clinically distinct groups of participants defined according to PGI-S ratings (‘no cough’; ‘very mild’/‘mild’; ‘moderate’; ‘severe’/‘very severe’) and Objective Cough Counts (0–33 percentile; 34–66 percentile; 67–100 percentile) were evaluated using between-group effect sizes estimates [47] (calculated by dividing mean differences by the pooled standard deviation for the reference group) at WK4. Effects sizes of 0.20, 0.50, and 0.80 were interpreted as small, moderate and large respectively [45, 46]. The statistical significance (p ≤ 0.05) of differences in scores between groups was calculated using a Kruskal-Wallis test. The expectation was that higher ratings of cough severity (PGI-S) and cough frequency (Awake Cough Count) would be associated with higher SCCD and Cough Severity VAS scores and lower LCQ scores. Large differences were hypothesized between groups defined according to PGI-S (PRO) scores and smaller differences hypothesized between groups defined according to objective Awake Cough Counts.
  - **Responsiveness:** The extent to which SCCD, LCQ and Cough Severity VAS scores were able to detect change was evaluated using data from BL to WK12. Subjects were categorised into ‘improved’, ‘no change’ and ‘worsened’ groups according to changes on the PGI-S (improved ≤-1, stable = 0, worsened ≥ 1), PGI-C (‘A little/Much/Very much better’ vs ‘No change’ vs ‘A little/Much/Very much worse’) and Awake Cough Count (improved ≤-30%, stable <|30%|, worsened ≥ 30%). Mean change scores were compared within groups via the calculation of within-group effect sizes (mean change score divided by the SD of the score at the earlier timepoint) [48] with the magnitude of effect sizes interpreted using similar criteria to those used in the known-group comparisons [45, 46]. Improvements in scores (i.e., lower scores for the SCCD and Cough Severity VAS and higher LCQ Total and Physical Domain scores) were anticipated among participants classified as improved on the anchor measures and these were expected to be greater than those classified as stable. Participants classified as worsened were hypothesised to show a worsening in PRO scores (i.e., higher SCCD and Cough Severity VAS scores and lower LCQ Total and Physical Domain scores). One-way analysis of variance (ANOVA) F-tests were employed to evaluate the statistical significance of any differences in change scores between groups classified as worsened, stable, and improved.

**Table 1:** Psychometric properties investigated, specific statistical analyses, and interpretation of results

Psychometric property	Time period	Target PRO	Reference measure(s)	Statistical procedure(s)	Interpretation
Sample demographic and clinical characteristics	Screening	N/A	N/A	Descriptive (mean, standard deviation, frequency, percentage)	Descriptive summary of sample
Quality of completion	BL, WK4 and WK12	SCCD, LCQ, Cough Severity VAS	N/A	Descriptive (frequency, percentage), stacked histogram, estimated reliability (Spearman-Brown)	Descriptive summary of missingness and estimated reliability for SCCD to inform number of items/days required for average scoring
Descriptive statistics	BL, WK4 and WK12	SCCD, LCQ, Cough Severity VAS	N/A	Descriptive (frequency, percentage)	Floor/ceiling effects (>15% participants) [39]
Internal consistency	WK4	LCQ Physical, LCQ Total	N/A	Cronbach’s alpha coefficient.	Reliability of items within multi-item scales. Cronbach’s alpha ≥ 0.70 considered acceptable [40]
Test-retest	Stability defined between WK2 and WK4	SCCD, LCQ, Cough Severity VAS	PGI-S, LCQ (Total), Cough Severity VAS, Awake Cough Count	Intraclass correlations ([2,1] and [2,k]) [41, 42]	Stability of scales over time when no change has occurred. ICC < 0.50 poor reliability, 0.50 and 0.75 moderate reliability, 0.75 and 0.90 good reliability, 0.90 excellent reliability [41]
Multi-trait (Item-scale) analysis	WK4	LCQ Total, LCQ Physical	N/A	Polyserial correlations	Dimensionality/construct validity through item-level correlations with defined scales (at least 0.40 and highest with own corrected domain) [43, 44]
Convergent validity analysis	WK4	SCCD	LCQ (Total, Physical, Psychological, Social), Cough Severity VAS, PGI-S, EQ-5D items and VAS, Awake Cough Count, Night Cough Count	Polyserial and Spearman correlations	Construct validity through hypothesized correlations with similar constructs. Moderate to high correlations (≥ 0.3) hypothesized with disease-specific measures (LCQ, Cough Severity VAS, PGI-S) and awake cough counts. Weak correlations (<0.3) hypothesized with generic measures (EQ-5D scores) and Night Cough Count. Criteria met if ≥ 75% of the results in accordance with predefined hypotheses [39]
Known groups comparisons	WK4	SCCD, LCQ, Cough Severity VAS	PGI-S, Awake Cough Count	Standardised between-group effect sizes, Kruskal-Wallis test	Construct validity through score differences between clinically known groups. Effects sizes of 0.20, 0.50, and 0.80 interpreted as small, moderate and large respectively [45, 46] Large differences hypothesized between groups defined according to PGI-S (PRO) scores. Smaller differences hypothesized between groups defined according to objective Awake Cough Count
Responsiveness	Change from BL to WK12	SCCD, LCQ, Cough Severity VAS	PGI-S, PGI-C Awake Cough Count	Descriptives (mean, standard deviation, median, minimum, maximum), standardised within-group effect size, One-way ANOVA F-test	Longitudinal validity/responsiveness through tracking a score when a change has in health state has occurred. Effects sizes of 0.20, 0.50, and 0.80 interpreted as small, moderate and large respectively [45, 46] Greatest improvements in PRO scores hypothesized among participants classified as ‘improved’. PRO scores expected to indicate worsening in those participants classified as ‘worsened’
Anchor-based score interpretation	Change from BL to WK12	SCCD, LCQ, Cough Severity VAS	PGI-S, PGI-C, Awake Cough Count	Polyserial correlations, Descriptives (within- and between-group mean, median, 95% confidence intervals, minimum, maximum, percentiles, frequency, percentage, percentile change), Receiver Operating Characteristic Curves, Empirical cumulative distribution function, Probability density function, discriminant analysis	Informs interpretation of scores at group- and individual-levels through reference to another score Thresholds identified via Max[sensitivity + specificity-1], i.e. Youden’s J index [50] and Min[(1 – sensitivity)^2^ +(1 - specificity) [2] i.e. the sum of squares method. Discriminant analysis was also used where the threshold at which the kernel densities intersected was taken as the estimate for MWPC [51]
Triangulation	Change from BL to WK12	SCCD, LCQ, Cough Severity VAS	PGI-S, PGI-C, Awake Cough Count	Correlation-weighted averages	Provides most appropriate point estimate from the range of meaningful change estimates

- **Interpretation of scores:** Thresholds to interpret MWPC for SCCD, LCQ and Cough Severity VAS scores were investigated via a series of anchor-based analyses to find the change score that optimally discriminates between two groups: ‘minimal/moderate/major improvement’ versus ‘stable’. Potential MWPC thresholds (i.e., all possible change scores) were evaluated by finding an optimal cut-point using receiver operating characteristic (ROC) curves, where sensitivity and specificity are valued equally. To define the MWPC thresholds two different methods [49] were considered to determine the threshold values: Max[sensitivity + specificity-1], i.e. Youden’s J index [50] and Min[(1 – sensitivity)^2^ +(1 - specificity)^2^] i.e. the sum of squares method. Discriminant analysis was also used where the threshold at which the kernel densities intersected was taken as the estimate for MWPC [51]. Primary analyses were based on data collected at baseline and WK12 and utilised the PGI-S (≥1 category improvement vs no change), PGI-C (‘A little/Much/Very much better’ vs ‘No change’) and Awake Cough Count (≥ 30% reduction vs < 30% reduction) as anchors. The suitability of proposed anchors was tested using a polyserial correlation coefficient to establish the relationship between the anchor and change in PRO score (anchors with correlations < 0.3 were not taken forward [52]). Triangulation was performed using a correlation-weighted average [53].

## Results

### Sample demographic and clinical characteristics

The analysis sample comprised of 310 participants (Table 2). The mean age of the sample was 59.1 years (SD = 11.79), with ages ranging from 19 to 81-years-old. The sample comprised of 77.7% female participants, with participants predominantly reporting their race as being White (87.1%) or Asian (11.9%). Most participants reported their time of RCC diagnosis as fewer than 10 years ago (56.8%).

**Table 2:** Participant socio-demographic and clinical characteristics

Demographic and clinical characteristic	Psychometric analysis population (N = 310)
Age (years)	
Mean (SD)	59.1 (11.79)
Median	61.0
Q1, Q3	51.0, 68.0
Min, Max	19, 81
Missing	0
Sex	
Male	69 (22.3%)
Female	241 (77.7%)
Missing	0
Race	
White	270 (87.1%)
Asian	37 (11.9%)
Native Hawaiian or Other Pacific Islander	1 (0.3%)
Not Reported	2 (0.6%)
Missing	0
Ethnicity	
Not Hispanic or Latino	300 (96.8%)
Hispanic or Latino	10 (3.2%)
Missing	0
History of chronic cough (time since diagnosis)	
< 10 years	176 (56.8%)
≥ = 10 years	134 (43.2%)
Missing	0
History of chronic cough (type of cough)	
Refractory Chronic Cough	139 (44.8%)
Unexplained Chronic Cough	171 (55.2%)
Missing	0

### SCCD cough frequency and cough severity

#### Quality of completion and descriptive statistics

Participant completion over a 7-day period (up to and including the respective study visit) at baseline, WK4, and WK12 was relatively high for the SCCD (83.9% - 91.3% missing no days or a single day). No floor and ceiling effects were observed at baseline for the SCCD Item 1 (Cough Frequency) and SCCD Item 5 (Cough Severity) scores.

#### Reliability

Test-retest reliability was good to excellent for the SCCD Item 1 (Cough Frequency) and SCCD Item 5 (Cough Severity) scores (ICC ≥ 0.887) among participants that were defined as stable between WK2 and WK4 according to PGI-S ratings and Awake Cough Count readings (Table 3).

**Table 3:** Internal consistency and test-retest reliability for select SCCD, LCQ and Cough Severity VAS scores

Score	No. of items	Cronbach’s alpha (WK4)	Test-retest ICC: WK2 and WK4—PGI-S (95% CI)*	Test-retest ICC: WK2 and WK4—awake cough count (95% CI)*
SCCD Item 1 (Frequency of cough)	1	n.a.	0.892	0.887
			(0.846–0.924)	(0.841–0.920)
			n = 142	n = 132
SCCD Item 5 (Severity of cough)	1	n.a.	0.917	0.896
			(0.881–0.941)	(0.853–0.926)
			n = 142	n = 132
LCQ Total	19	0.939	0.886	0.866
			(0.825–0.923)	(0.811–0.905)
			n = 144	n = 136
LCQ Physical domain	8	0.806	0.851	0.848
			(0.789–0.894)	(0.790–0.890)
			n = 144	n = 136
Cough Severity VAS	1	n.a.	0.952	0.935
			(0.925–0.968)	(0.900–0.957)
			n = 140	n = 129

* SCCD and Cough Severity VAS Weekly Averages used ICC [2,k] based on a multiple measurement, absolute agreement, two-way mixed effects model; LCQ analysis used ICC [2,1] based on a single measurement, absolute agreement, two-way mixed effects model [41, 42]

#### Convergent validity

High to very large correlations were observed between SCCD Item 5 (Cough Severity) with Cough Severity VAS (r = 0.685) and PGI-S (r = 0.759) and a moderate correlation observed between SCCD Item1 (Cough Frequency) with Awake Cough Count (r = 0.432). Convergent validity correlations between SCCD scores and LCQ and EQ-5D-5 L scores were consistent with or higher than hypothesized correlations in > 75% of instances (Table 4).

**Table 4:** Convergent validity correlations for SCCD Item 1 (Cough Frequency) and SCCD Item 5 (Cough Severity) at WK4

Convergent measure	SCCD Item 1 Frequency of cough weekly average (r_s_)	SCCD Item 5 Severity of cough weekly average (r_s_)
LCQ Total Score	−0.645^a^	−0.615^a^
LCQ Physical domain	−0.547^a^	−0.509^a^
LCQ Psychological domain	−0.626^a^	−0.605^a^
LCQ Social domain	−0.618^a^	−0.594^a^
Cough Severity VAS Weekly Score	0.656^a^	0.685^a^
PGI-S (Severity of Cough)	0.757^a^	0.759^a^
EQ-5D-5 L Mobility	0.084^b^	0.103^b^
EQ-5D-5 L Self-care	−0.064^b^	0.038^b^
EQ-5D-5 L Usual Activities	0.255^b^	0.270^b^
EQ-5D-5 L Pain/Discomfort	0.417^b^	0.393^b^
EQ-5D-5 L Anxiety/Depression	0.251^b^	0.322^b^
EQ-5D-5 L VAS	−0.061^b^	0.001^b^
Awake Cough Count^	0.432^a^	0.411^b^
Night Cough Count^	0.221^b^	0.266^b^

**^**Awake Cough Count: Measures of cough count based on those recorded during awake periods only; Night Cough Count: Measures of cough count based on those recorded during night periods only

^a^ Moderate to high correlation hypothesized (≥ 0.3)

^b^ Weak correlation hypothesized (< 0.3)

#### Known groups comparisons

**Fig. 2:**
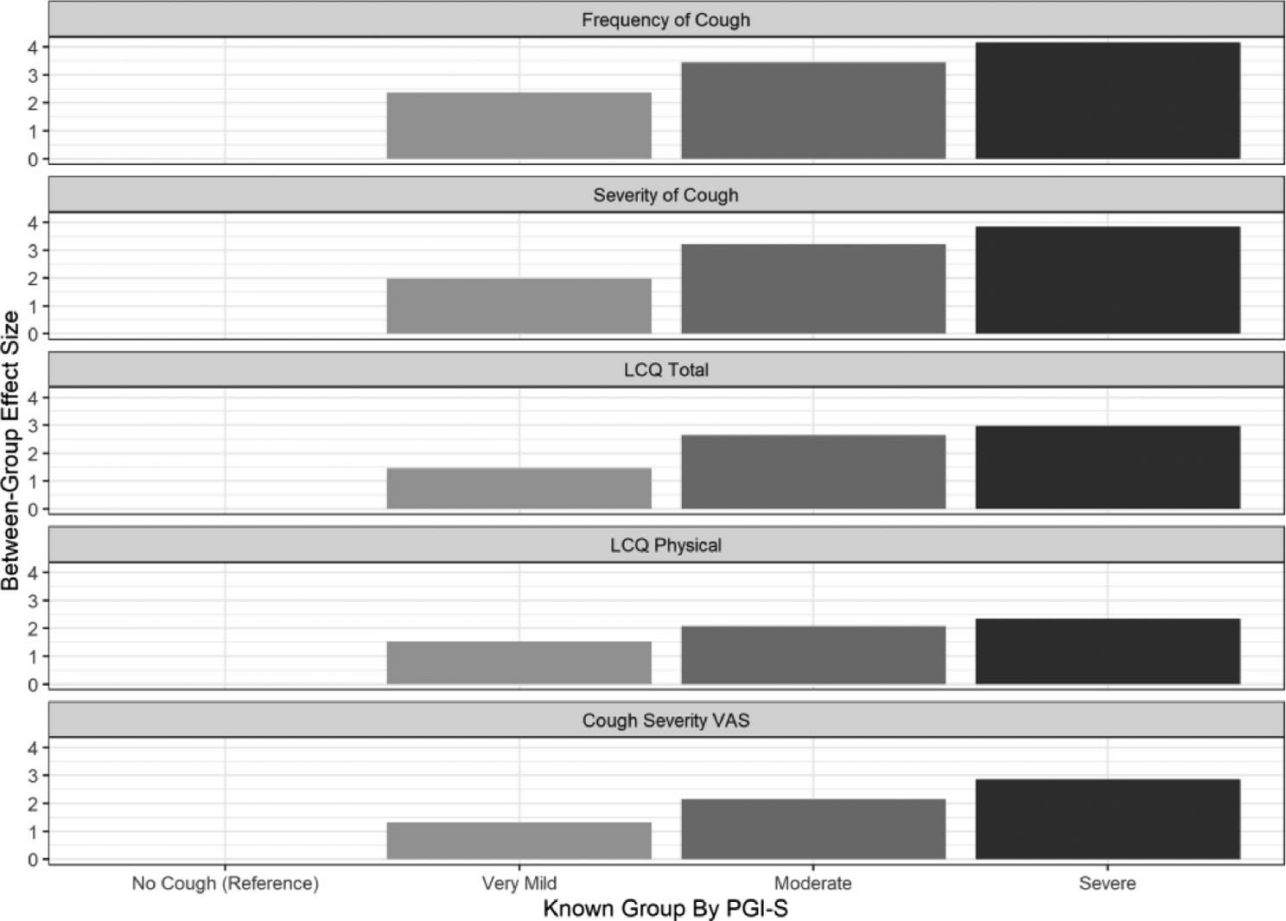
Known groups validity for SCCD Item 1 (Cough Frequency), SCCD Item 5 (Cough Severity), LCQ Total, LCQ Physical domain and Cough Severity VAS scores according to PGI-S ratings and Objective Cough Counts at WK4

Anticipated linear trends in group means were observed for SCCD Item 1 (Cough Frequency) and SCCD Item 5 (Cough Severity) scores among groups categorized according to PGI-S ratings and objective Awake Cough Counts (Fig. 2). For all scores, differences between groups defined according to PGI-S ratings were large (effects sizes 1.99–4.16) in accordance with pre-specified hypotheses. Smaller differences between groups defined according to Awake Cough Count were observed as expected (effect sizes ranging from small to large; 0.40–1.04). All differences between groups were statistically significant (p < 0.001).

#### Responsiveness

Ability to detect improvement was supported with the greatest mean change scores and large effect sizes observed for SCCD scores among participants classified as ‘improved’ according to PGI-S ratings (Table 5). Similar results were observed when change was defined according to PGI-C ratings and Awake Cough Count (see Supplementary Materials I). Statistically significant differences (p < 0.001) in mean change for SCCD Item 1 (Cough Frequency) and SCCD Item 5 (Cough Severity) scores were observed between ‘improved’, ‘stable’ and ‘worsened’ groups defined according to all external measures.

**Table 5:** Ability to detect change according to changes in PGI-S anchor ratings between BL and WK12

**PRO Score (range)** PGI-S anchor groups	n	Mean change score (SD)	Median change score (Min-Max)	Within groups effect size	Between Groups p-value
**SCCD Item 1—cough frequency (0–4)**					
Improved: change score ≤ = − 1	140	−1.08 (0.75)	−1.00 (−3.0, 0.4)	−2.15	<.001
Stable: change score = 0	92	−0.42 (0.54)	−0.33 (−1.9, 1.1)	−0.83	
Worsened: change score ≥ = 1	23	0.25 (0.94)	0.17 (−1.5, 2.8)	0.35	
**SCCD Item 5 – Cough Severity (0–4)**					
Improved: change score ≤ = − 1	140	−1.02 (0.66)	−1.00 (−3.0, 0.6)	−1.96	<.001
Stable: change score = 0	92	−0.34 (0.52)	−0.29 (−1.7, 1.0)	−0.61	
Worsened: change score ≥ = 1	23	0.20 (1.05)	0.02 (−1.7, 3.1)	0.28	
**LCQ Total (3–21)**					
Improved: change score ≤ = − 1	151	3.98 (3.48)	3.64 (−4.0, 14.3)	1.49	<.001
Stable: change score = 0	94	1.28 (1.96)	0.99 (−2.6, 7.6)	0.47	
Worsened: change score ≥ = 1	23	−0.16 (3.18)	0.45 (−7.1, 6.7)	−0.04	
**LCQ Physical (1–7)**					
Improved: change score ≤ = − 1	151	1.12 (1.00)	1.00 (−0.9, 3.9)	1.30	<.001
Stable: change score = 0	94	0.36 (0.70)	0.38 (−1.5, 2.4)	0.38	
Worsened: change score ≥ = 1	23	−0.18 (0.85)	−0.13 (−2.1, 1.1)	−0.17	
**Cough Severity VAS (0–100)**					
Improved: change score ≤ = − 1	138	−33.62 (23.92)	−33.97 (−92.4, 13.1)	−2.16	<.001
Stable: change score = 0	93	−10.45 (14.22)	−8.86 (−69.1, 24.3)	−0.71	
Worsened: change score ≥ = 1	21	−0.10 (15.20)	3.05 (−41.2, 35.0)	−0.00	

Within groups effect size: mean change score divided by the SD of the score at Baseline

Between groups p-value: F-test from a one-way ANOVA

### Interpretation of scores

Correlations between changes in SCCD Item 1 (Cough Frequency) and SCCD Item 5 (Cough Severity) with selected anchors (PGI-S, PGI-C, and Awake Cough Count) between BL and WK12 were all acceptable (i.e., r ≥ 0.3). Estimates of MWPC for SCCD Item 1 (Cough Frequency) and SCCD Item 5 (Cough Severity) based on interpretation of ROC curves and discriminant analysis are outlined in Table 6. Area under the curve (AUC) values for SCCD Item 1 (Cough Frequency) were 0.753 (PGI-S), 0.800 (PGI-C) and 0.708 (Awake Cough Count); for SCCD Item 5 (Cough Severity) they were 0.787 (PGI-S), 0.778 (PGI-C) and 0.715 (Awake Cough Count). The triangulated threshold for SCCD Item 1 (Cough Frequency) was − 0.82 (95% CI [−0.93, − 0.70]) and for SCCD Item 5 (Cough Severity) was − 0.69 (95% CI [−0.71, − 0.66]).

**Table 6:** Anchor-based MWPC threshold estimates and anchor-scale change score correlations between BL and WK12 for SCCD Item 1 (Cough Weekly Average, SCCD Item 5 Weekly Average, LCQ Total, LCQ Physical domain, and Cough Severity VAS Weekly Average)

PRO score (range)	MWPC estimates (correlation-weighted average with 95% CIs in bold)	Anchor polyserial correlations
SCCD Item 1 (Cough Frequency) Weekly Average (0–4)	−0.8 (PGI-S)^1^ −0.6 (PGI-C)^1^ −1.0 (Awake Cough Count)^1^ −0.8 (PGI-S)^2^ −0.8 (PGI-C)^2^ −1.0 (Awake Cough Count)^2^ **−0.82 [−0.93, −0.70]**	0.6 (PGI-S) 0.6 (PGI-C) 0.5 (Awake Cough Count)
SCCD Item 5 (Cough Severity) Weekly Average (0–4)	−0.7 (PGI-S)^1^ −0.7 (PGI-C)^1^ −0.7 (Awake Cough Count)^1^ −0.7 (PGI-S)^2^ −0.6 (PGI-C)^2^ −0.7 (Awake Cough Count)^2^ **−0.69 [−0.71, −0.66]**	0.7 (PGI-S) 0.6 (PGI-C) 0.5 (Awake Cough Count)
LCQ Total (3–21)	2.1 (PGI-S)^1^ 1.8 (PGI-C)^1^ 1.8 (Awake Cough Count)^1^ 3.2 (PGI-S)^2^ 2.6 (PGI-C)^2^ 2.8 (Awake Cough Count)^2^ **2.36 [1.92, 2.81]**	−0.6 (PGI-S) −0.7 (PGI-C) −0.6 (Awake Cough Count)
LCQ Physical (1–7)	0.8 (PGI-S)^1^ 0.5 (PGI-C)^1^ 0.8 (Awake Cough Count)^1^ 0.9 (PGI-S)^2^ 0.7 (PGI-C)^2^ 0.9 (Awake Cough Count)^2^ **0.77 [0.64, 0.89]**	−0.6 (PGI-S) −0.6 (PGI-C) −0.6 (Awake Cough Count)
Cough Severity VAS (0–100)	−20.2 (PGI-S)^1^ −9.3 (PGI-C)^1^ −17.3 (Awake Cough Count)^1^ −26.4 (PGI-S)^2^ −14.8 (PGI-C)^2^ −21.9 (Awake Cough Count)^2^ **−17.73 [−22.95, −12.51]**	0.6 (PGI-S) 0.7 (PGI-C) 0.5 (Awake Cough Count)

Correlations were z-transformed prior to their use in the correlation-weighted average

^1^ = estimates derived from ROC curve analyses; ^2^ = estimates derived from discriminant analyses

### LCQ total and physical domain score

#### Quality of completion and descriptive statistics

High levels of completion (86.5% - 100%) were observed for the LCQ at baseline, WK4 and WK12. No floor and ceiling effects were observed at baseline for the LCQ Total and Physical domain scores.

#### Reliability

Internal consistency reliability was supported for both the LCQ Total and Physical domain scores with Cronbach’s alpha values at WK4 of 0.939 and 0.806 respectively. Test-retest reliability was good to excellent for the LCQ Total and Physical domain scores (ICC ≥ 0.848) among participants that were defined as stable between WK3 and WK4 according to PGI-S ratings and Awake Cough Count readings (Table 3).

#### Multi-trait (Item-scale) analysis

For the LCQ Physical domain score, acceptable item-scale correlations were observed for all items except for Item 15 (‘Over the last 2 weeks, have you had a lot of energy?’; r = 0.36). With the exception of Item 15 and Item 11 (‘Over the last 2 weeks, how many times a day have you had coughing bouts?’), correlation coefficients were highest with their own corrected domain than with any other domain of the LCQ (Table 7).

**Table 7:** Multi-trait (item-scale) analysis for LCQ at WK4

LCQ Item	Polyserial correlation (corrected for overlap) with domain scores (N = 291)		
	LCQ Physical domain	**LCQ Domain** Psychological domain	LCQ Social domain
Item 1: Chest/Stomach Pains Result Cough	**0.674**	0.464	0.496
Item 2: Bothered by Sputum When You Cough	**0.433**	0.421	0.427
Item 3: Tired Because of Your Cough	**0.735**	0.625	0.683
Item 4: Felt in Control of Your Cough	0.412	**0.601**	0.583
Item 5: Embarrassed by Your Coughing	0.576	**0.818**	0.819^b^
Item 6: Cough Made Me Anxious	0.674	**0.770**	0.719
Item 7: Cough Interfered With My Job	0.750^b^	0.704	**0.724**
Item 8: Cough Interfered Enjoyment of Life	0.728	0.795	**0.822**
Item 9: Exposure Paints/Fumes Made Cough	**0.463**	0.320	0.365
Item 10: Has Cough Disturbed Your Sleep	**0.628**	0.491	0.570
Item 11: How Many Times had Coughing Bouts	**0.602**	0.728^b^	0.712^b^
Item 12: Cough Has Made Me Feel Frustrated	0.633	**0.854**	0.818
Item 13: Cough Has Made Me Feel Fed Up	0.684	**0.819**	0.781
Item 14: Suffered Hoarse Voice Result Cough	**0.513**	0.375	0.402
Item 15: Have You Had a Lot of Energy	**0.360**^**a**^	0.388^b^	0.396^b^
Item 16: Worried Cough Serious Illness	0.578	**0.631**	0.577
Item 17: Concerned People Think	0.544	**0.790**	0.788
Item 18: My Cough Interrupted Conversation	0.604	0.743^b^	**0.738**
Item 19: My Cough Annoyed Family/Friends	0.496	0.772^b^	**0.674**

Total N represents all participants with non-missing score on any LCQ item at WK4

Polyserial correlations used between continuous and ordinal variables

Item-scale correlation corrected for overlap (relevant item removed from its scale for correlation). Hypothesized to be highest in same row

^a^ Less than desirable item convergent validity: item correlation with its own scale is significantly less than r = 0.4

^b^ Less than desirable item discriminant validity: item correlation with competing scale is higher than the correlation with its own scale

Correlation values made bold to identify which items relate to which scales

#### Known-groups comparisons

Anticipated linear trends in group means were observed for LCQ Total and LCQ Physical scores among groups categorized according to PGI-S ratings and objective Awake Cough Counts (Fig. 2). For all scores, differences between groups defined according to PGI-S ratings were large (effects sizes 1.46–2.96) in accordance with pre-specified hypotheses. Smaller differences between groups defined according to Awake Cough Count were observed as expected (small to large effect sizes; 0.47–0.98). All differences between groups were statistically significant (p < 0.001).

#### Responsiveness

Ability to detect improvement was supported with the greatest mean change scores and large effect sizes observed for LCQ scores among participants classified as ‘improved’ according to PGI-S ratings (Table 5). Similar results were observed when change was defined according to PGI-C ratings and Awake Cough Count (see Supplementary Materials I). Statistically significant differences (p < 0.001) in LCQ Total and LCQ Physical domain mean change scores were observed between ‘improved’, ‘stable’ and ‘worsened’ groups defined according to all external measures.

#### Interpretation of scores

Correlations between changes in LCQ Total and LCQ Physical domain scores with selected anchors (PGI-S, PGI-C, and Awake Cough Count) between BL and WK12 were all acceptable (i.e., r ≥ 0.3). Estimates of MWPC for LCQ Total and LCQ Physical domain scores, based on interpretation of ROC curves and discriminant analysis are outlined in Table 6. AUC values for LCQ Total were 0.745 (PGI-S), 0.785 (PGI-C) and 0.750 (Awake Cough Count); for LCQ Physical they were 0.730 (PGI-S), 0.767 (PGI-C) and 0.732 (Awake Cough Count). The triangulated threshold for the LCQ Total score was 2.36 (95% CI [1.92, 2.81]) and for the LCQ Physical domain score was 0.77 (95% CI [0.64, 0.89]).

### Cough severity VAS

The cough severity VAS demonstrated acceptable measurement properties (test-retest reliability, known-groups validity and responsiveness) in accordance with predefined criteria. The triangulated threshold for MWPC for the Cough Severity VAS was − 17.73 (95% CI [−22.95, − 12.51]). Further details are provided in Supplementary Materials II.

## Discussion

### SCCD psychometric properties

This study provides strong evidence for the reliability, validity and responsiveness of SCCD Item 1 (Cough Frequency) and Item 5 (Cough Severity) scores. This supplements prior qualitative research confirming the content validity of the SCCD scores [32], supporting these measures as ‘fit-for-purpose’ to assess endpoints regarding patient-reported improvements in cough frequency and cough severity in RCC clinical trials in accordance with regulatory expectations and scientific standards [11–13, 33].

Objective cough frequency and monitoring is commonly used to assess primary endpoints in clinical trials. However, it is acknowledged that cough is both an objectively observable pathophysiological phenomenon and a symptom experienced by patients [54]. Objective and subjective assessments of cough frequency likely capture distinct aspects of patients’ experience of RCC [38]. This is evident in the current study via the modest correlations observed between SCCD Item 1 (Cough Frequency) and Item 5 (Cough Severity) scores and objective measures of cough, a finding consistent with wealth of prior research [54]. Daily assessment of cough frequency and cough severity via PRO measures such as the SCCD, therefore, are critical for determining effects of treatments on the underlying symptomology of cough and important information on how patients feel and function [21]. Such measures can also be used to provide additional insights into the lived experience of RCC patients given limitations of existing cough monitors (including the VitaloJAK) that are typically only used within isolated 24 hour periods due to the burden they can pose to patients and the expense associated with monitoring over longer time periods [55].

### LCQ psychometric properties

Findings from the current study are consistent with other recent studies supporting the reliability, validity and responsiveness of LCQ Total and Physical domain scores in this context of use [18, 20]. Levels of missing data for the LCQ Total and Physical domain scores were low and no floor or ceiling effects in associated scores were observed. Consistent with prior research exploring the measurement properties of the LCQ in RCC populations [18, 20], supportive evidence of reliability (internal consistency and test-retest reliability), construct validity (including convergent validity and known-groups comparisons) and responsiveness was observed [18, 20]. Of note, reported test-retest reliability ICCs in this study were higher than in prior exploration of the measurement properties of the LCQ Total and Physical Domain scores [18, 20] likely owing to the use of the PGI-S, rather than the PGI-C to define stability and a larger number of ‘stable’ participants identified (n = 136–144 vs n = 32–61) resulting in more robust estimates. The triangulated threshold estimate for the LCQ Total score MWPC of 2.36 (95% CI 1.92, 2.81) derived from the current study slightly exceeds those reported previously for the LCQ Total score (1.3 to 2.3 in previous analyses) [20, 56, 57]. Prior anchor-based explorations of MWPC for the LCQ Total score have been based on use of a single anchor measure – the PGI-C [20, 56, 57] – for which considerable limitations have been noted (e.g., reliance on recall) [58]. By contrast, the current study made use of multiple subjective and objective anchors including the both patient global assessment instruments: e.g. the PGI-C and the PGI-S. Specifically, the PGI-S is a static assessment of disease severity. Its validity has been confirmed with chronic cough populations [59], it is also generally preferred to comparative assessments, and objective assessments including awake cough count. The use of multiple anchors in this manner is generally recommended to account for potential limitations in anchors and their relationship to the PRO scores of interest [60].

### Cough severity VAS

Findings from the current study are consistent with other recent studies supporting the reliability, validity and responsiveness of the Cough Severity VAS in this context of use [23, 61]. Levels of missing data for the Cough Severity VAS scores were low and no floor or ceiling effects in associated scores were observed. Consistent with prior research exploring the measurement properties of Cough Severity VAS in RCC populations, supportive evidence of reliability (test-retest reliability), construct validity (known-groups comparisons) and responsiveness was observed [23]. Test-retest reliability ICCs were indicative of excellent reliability and surpassed the performance of a similar measure which demonstrated only moderate reliability (ICC = 0.51) [23], again likely owing to better identification of stable participants via the PGI-S and more robust sample sizes for ‘stable’ participants in this study. In research conducted by Nguyen et al (2021) [23] two potential definitions of MWPC thresholds for a change in Cough Severity VAS were reported; a ≥ 20-mm or a ≥ 30-mm threshold, with these thresholds being reflective of participants reporting themselves as at least minimally or much improved on the PGI-C respectively. In the current study, a triangulated threshold of − 17.7 (95% CI − 22.95, − 12.51) for the Cough Severity VAS was derived based on consideration of multiple subjective and objective anchors.

### Strengths and limitations

Prior exploration of the measurement properties of cough diaries, the LCQ and Cough Severity VAS have been based predominantly on data collected from patients from select English-speaking countries [20, 23, 26]. As such, evaluation of PRO data collected from the PAGANINI study (a global multinational study in which participants reflecting the global population of RCC patients were recruited from 19 countries across North America, South America, Europe and Australia) [29] is a key strength of the reported analyses.

The use of multiple anchor-measures for evaluating MWPC, including the use of static assessments of health status (i.e., PGI-S) which were used considering best practice and regulatory (FDA) recommendations, is also a strength of this study [33]. While a primary goal of clinical studies is to ‘improve’ cough severity, there is a limited understanding of thresholds of MWPC that would be indicative of worsening, and which may be valuable for disease monitoring and guiding decisions in clinical practice. Generally, it cannot be assumed that thresholds of MWPC as established for improvement in this study, apply to worsening as well [62]. Clinical trial eligibility criteria which require participants to be experiencing cough at baseline that is of at least moderate severity, the administration of investigational therapy and placebo responses commonly observed in chronic cough clinical trials minimise the likelihood of experiencing worsening in this context. Accordingly, the proportion of participants classified as ‘worsened’ in the current study was not sufficient for exploring such estimates. This is a limitation and remains a topic for future research.

### Conclusions

Assessment of the frequency and severity of RCC symptoms and the impact of these symptoms on patients’ lives is important for evaluating the efficacy of interventions for RCC in clinical trials. Findings from the current study demonstrate that items from the newly developed SCCD are reliable, valid and responsive measures of cough frequency and severity for use in multinational clinical trials. This research complements existing research supporting the use of the LCQ and Cough Severity VAS in this context.

## Electronic supplementary material

Below is the link to the electronic supplementary material.

Supplementary Material 1

## Data Availability

The datasets generated and/or analysed during the current study are not publicly available. Bayer’s position on sharing clinical trial data with qualified researchers is outlined in https://clinicaltrials.bayer.com/transparency-policy/.

## Abbreviations

ANOVA: Analysis of variance
AUC: Area under the curve
CI: Confidence interval
CSD: Cough Severity Diary
EMA: European Medicines Agency
ICC: Intraclass correlation coefficient
FDA: Food and Drug Administration
HRQoL: Health-related quality of life
LCQ: Leicester Cough Questionnaire
MWPC: Meaningful within-patient change
PGI-C: Patient Global Impression of Change
PGI-S: Patient Global Impression of Severity
PRO: Patient-reported outcome
RCC: Refractory chronic cough
ROC: Receiver operating characteristic
SCCD: Severity of Chronic Cough Diary
SD: Standard deviation
VAS: Visual analogue scale
VRS: Verbal rating scale
WK: Week

## References

[1] Morice AH, Millqvist E, Bieksiene K, et al. (2020) ERS guidelines on the diagnosis and treatment of chronic cough in adults and children. Eur Respir J 55(1). 10.1183/13993003.01136-2019 PMC6942543 31515408

[2] Song W-J, Chang Y-S, Faruqi S, et al. (2015) The global epidemiology of chronic cough in adults: a systematic review and meta-analysis. Eur Respir J 45(5):1479–1481 25657027 10.1183/09031936.00218714

[3] Puente-Maestu L, Dávila I, Quirce S, et al. (2023) Burden of refractory and unexplained chronic cough on patients’ lives: a cohort study. ERJ Open Res 9(5). 10.1183/23120541.00425-2023 PMC10518856 37753282

[4] Kubo T, Tobe K, Okuyama K, et al. (2021) Disease burden and quality of life of patients with chronic cough in Japan: a population-based cross-sectional survey. BMJ Open Respir Res 8(1). 10.1136/bmjresp-2020-000764 PMC8011713 33785505

[5] Morice A, Dicpinigaitis P, McGarvey L, Birring SS (2021) Chronic cough: new insights and future prospects. Eur Respir Rev 30 (162). 10.1183/16000617.0127-2021 PMC9488126 34853095

[6] Young EC, Smith JA (2010) Quality of life in patients with chronic cough. Ther Adv Respir Dis 4(1):49–55 20051447 10.1177/1753465809358249

[7] Abdulqawi R, Dockry R, Holt K, et al. (2015) P2×3 receptor antagonist (AF-219) in refractory chronic cough: a randomised, double-blind, placebo-controlled phase 2 study. Lancet 385(9974):1198–1205 25467586 10.1016/S0140-6736(14)61255-1

[8] Muccino DR, Morice AH, Birring SS, et al. (2020) Design and rationale of two phase 3 randomised controlled trials (COUGH-1 and COUGH-2) of gefapixant, a P2×3 receptor antagonist, in refractory or unexplained chronic cough. ERJ Open Research 6(4). 10.1183/23120541.00284-2020 PMC7682670 33263037

[9] Smith JA, Kitt MM, Morice AH, et al. (2020) Gefapixant, a P2×3 receptor antagonist, for the treatment of refractory or unexplained chronic cough: a randomised, double-blind, controlled, parallel-group, phase 2b trial. Lancet Respir Med 8(8):775–785 32109425 10.1016/S2213-2600(19)30471-0

[10] Irwin RS, Baumann MH, Bolser DC, et al. (2006) Diagnosis and management of cough executive summary: ACCP evidence-based clinical practice guidelines. Chest 129(1). 10.1378/chest.129.1_suppl.1S PMC3345522 16428686

[11] US Food & Drug Administration. Guidance for industry: patient-reported outcome measures: use in medical product development to support labeling claims. 2009. 10.1186/1477-7525-4-79 PMC1629006 17034633

[12] Food and Drug Administration (2022) Patient-focused drug development: selecting, developing, or modifying fit-for-purpose clinical outcome assessments. Guidance Industry. draft. https://www.fda.gov/media/159500/download. 10.1016/j.jval.2023.04.006 37116698

[13] Committee for Medicinal Products for Human Use (2005) Reflection paper on the regulatory guidance for the use of health-related quality of life (HRQL) measures in the evaluation of medicinal products. London: European Medicines Agency.

[14] Vernon M, Leidy NK, Nacson A, Nelsen L (2009) Measuring cough severity: perspectives from the literature and from patients with chronic cough. Cough 5:1–8 19298650 10.1186/1745-9974-5-5 PMC2669040

[15] Spinou A, Birring SS (2014) An update on measurement and monitoring of cough: what are the important study endpoints? J Thoracic Dis 6 (Suppl 7). 10.3978/j.issn.2072-1439.2014.10.08 PMC4222923 25383207

[16] La Orden Abad M D, Haberland C, Filonenko A, et al. (2021) Understanding patients’ experience of chronic cough. In: TP104 TP104 Understanding mechanisms and outcomes observed in pulmonary rehabilitation. American Thoracic Society, pp A4160–A4160.

[17] Domingo C, Fernandez M, Garin N, et al. (2023) Determining what represents value in the treatment of refractory or unexplained chronic cough from the perspective of key stakeholders in Spain using multi-criteria decision analysis. Appl Health Econ Health Pol 21(1):119–130 10.1007/s40258-022-00770-9 PMC9628572 36319945

[18] Birring S, Prudon B, Carr A, Singh S, Morgan M, Pavord I (2003) Development of a symptom specific health status measure for patients with chronic cough: leicester Cough Questionnaire (LCQ). Thorax 58(4):339–343 12668799 10.1136/thorax.58.4.339 PMC1746649

[19] Yousaf N, Lee KK, Jayaraman B, Pavord ID, Birring SS (2011) The assessment of quality of life in acute cough with the Leicester Cough Questionnaire (LCQ-acute). Cough 7(1):1–5 21767404 10.1186/1745-9974-7-4 PMC3169450

[20] Nguyen AM, Schelfhout J, Muccino D, et al. (Jan-Dec 2022) Leicester Cough Questionnaire validation and clinically important thresholds for change in refractory or unexplained chronic cough. Ther Adv Respir Dis. 16:17534666221099737. 10.1177/17534666221099737. 10.1177/17534666221099737 PMC9149626 35614875

[21] Kum E, Guyatt GH, Munoz C, et al. (2022) Assessing cough symptom severity in refractory or unexplained chronic cough: findings from patient focus groups and an international expert panel. ERJ Open Res 8(1). 10.1183/23120541.00667-2021 PMC8918938 35295233

[22] Birring S, Brightling C, Symon F, Barlow S, Wardlaw A, Pavord I (2003) Idiopathic chronic cough: association with organ specific autoimmune disease and bronchoalveolar lymphocytosis. Thorax 58(12):1066–1070 14645977 10.1136/thorax.58.12.1066 PMC1746533

[23] Martin Nguyen A, Bacci ED, Vernon M, et al. (2021) Validation of a visual analog scale for assessing cough severity in patients with chronic cough. Ther Adv Respir Dis 15:17534666211049743 34697975 10.1177/17534666211049743 PMC8552382

[24] González-Fernández M, Ghosh N, Ellison T, McLeod JC, Pelletier CA, Williams K (2014) Moving beyond the limitations of the visual analog scale for measuring pain: novel use of the general labeled magnitude scale in a clinical setting. Am J Phys Med Rehabil 93(1):75–81 23900013 10.1097/PHM.0b013e31829e76f7

[25] Vernon M, Kline Leidy N, Nacson A, Nelsen L (2010) Measuring cough severity: development and pilot testing of a new seven-item cough severity patient-reported outcome measure. Ther Adv Respir Dis 4(4):199–208 20519373 10.1177/1753465810372526

[26] Martin Nguyen A, Bacci E, Dicpinigaitis P, Vernon M (2020) Quantitative measurement properties and score interpretation of the Cough Severity Diary in patients with chronic cough. Ther Adv Respir Dis 14:1753466620915155 32345170 10.1177/1753466620915155 PMC7225816

[27] Birring SS, Dicpinigaitis PV, Smith JA, et al. (2023) Efficacy and safety of gefapixant for refractory or unexplained chronic cough over 52 weeks. Am J Respir Crit Care Med 207(11):1539–1542 36996347 10.1164/rccm.202211-2128LE PMC10263136

[28] McGarvey L, Sher M, Shvarts YG, et al. (2023) The efficacy and safety of gefapixant in a Phase 3b trial of patients with recent-onset Chronic Cough. Lung 201(2):111–118 36879087 10.1007/s00408-023-00606-w PMC10115701

[29] Dicpinigaitis PV, Morice AH, Smith JA, et al. (Jun 2023) Efficacy and safety of eliapixant in refractory chronic cough: the randomized, placebo-controlled Phase 2b PAGANINI study. Lung 201(3):255–266.10.1007/s00408-023-00621-x 10.1007/s00408-023-00621-x PMC10234230 37261531

[30] Patrick DL, Burke LB, Gwaltney CJ, et al. (2011) Content validity—establishing and reporting the evidence in newly developed patient-reported outcomes (PRO) instruments for medical product evaluation: ISPOR PRO good research practices task force report: part 1—eliciting concepts for a new PRO instrument. Value Health 14(8):967–977 22152165 10.1016/j.jval.2011.06.014

[31] Patrick DL, Burke LB, Gwaltney CJ, et al. (2011) Content validity—establishing and reporting the evidence in newly developed patient-reported outcomes (PRO) instruments for medical product evaluation: ISPOR PRO Good Research Practices Task Force report: part 2—assessing respondent understanding. Value Health 14(8):978–988 22152166 10.1016/j.jval.2011.06.013

[32] de la Orden Abad M, Haberland C, Karn H, Skalicky A, Hareendran A (2023) The Severity of Chronic Cough Diary (SCCD): development and content validation of a novel patient-reported outcome instrument for evaluating the symptom experience of chronic cough. J Patient-reported Outcomes 7(1):65 10.1186/s41687-023-00605-8 PMC10333155 37428359

[33] Food and Drug Administration. Patient-focused drug development: incorporating clinical outcome assessments into endpoints for regulatory decision-making. Draft guidance for industry, food and drug administration staff, and other stakeholders. https://www.fda.gov/media/166830/download. 2023.

[34] Smith JA, Holt K, Dockry R, et al. (2021) Performance of a digital signal processing algorithm for the accurate quantification of cough frequency. Eur Respir J 58(2). 10.1183/13993003.04271-2020 33875494

[35] Raj A, Pavord D, Birring S (2009) Clinical cough IV: what is the minimal important difference for the Leicester Cough Questionnaire? Pharmacol Therapeutics Cough 311–320. 10.1007/978-3-540-79842-2_16 18825348

[36] Herdman M, Gudex C, Lloyd A, et al. (2011) Development and preliminary testing of the new five-level version of EQ-5D (EQ-5D-5L). Qual Life Res 20:1727–1736 21479777 10.1007/s11136-011-9903-x PMC3220807

[37] Mines D, Bacci E, Nguyen AM, Shaffer S, Smith J, Vernon M (2019) Assessment of inter-and intra-rater reliability of objective cough frequency in patients with chronic cough. Eur Respir Soc.

[38] Schelfhout J, Nguyen AM, Birring SS, et al. (2022) Validation and meaningful change thresholds for an objective cough frequency measurement in chronic cough. Lung 200(6):717–724 36348054 10.1007/s00408-022-00587-2 PMC9675653

[39] Terwee CB, Bot SD, de Boer MR, et al. (2007) Quality criteria were proposed for measurement properties of health status questionnaires. J Clin Epidemiol 60(1):34–42 17161752 10.1016/j.jclinepi.2006.03.012

[40] Nunnally J, Bernstein I (1994) Psychometric Theory, 3rd edn. MacGraw-Hill, New York

[41] Koo TK, Li MY (2016) A guideline of selecting and reporting intraclass correlation coefficients for reliability research. J Cchiropractic Medi 15(2):155–163 10.1016/j.jcm.2016.02.012 PMC4913118 27330520

[42] Qin S, Nelson L, McLeod L, Eremenco S, Coons SJ (2019) Assessing test–retest reliability of patient-reported outcome measures using intraclass correlation coefficients: recommendations for selecting and documenting the analytical formula. Qual Life Res 28:1029–1033 30547346 10.1007/s11136-018-2076-0 PMC6439259

[43] Campbell DT, Fiske DW (1959) Convergent and discriminant validation by the multitrait-multimethod matrix. Psychol Bull 56(2):81 13634291

[44] Fayers PM, Machin D (2013) Quality of life: the assessment, analysis and interpretation of patient-reported outcomes. John Wiley & Sons

[45] Chassany O, Sagnier P, Marquis P, Fullerton S, Aaronson N, Group ERIoQo LA (2002) Patient-reported outcomes: the example of health-related quality of life—a European guidance document for the improved integration of health-related quality of life assessment in the drug regulatory process. Drug Inf J 36(1):209–238

[46] Cohen J (2013) Statistical power analysis for the behavioral sciences. Academic press

[47] Hedges LV (1981) Distribution theory for Glass’s estimator of effect size and related estimators. Journal of Educational Statistics 6(2):107–128

[48] Kazis LE, Anderson JJ, Meenan RF (1989) Effect sizes for interpreting changes in health status. Med Care S178–S189. 10.1097/00005650-198903001-00015 2646488

[49] Froud R, Abel G (2014) Using ROC curves to choose minimally important change thresholds when sensitivity and specificity are valued equally: the forgotten lesson of pythagoras. theoretical considerations and an example application of change in health status. PLoS One 9 (12). 10.1371/journal.pone.0114468 PMC4256421 25474472

[50] Youden WJ (1950) Index for rating diagnostic tests. Cancer 3(1):32–35 15405679 10.1002/1097-0142(1950)3:1<32::aid-cncr2820030106>3.0.co;2-3

[51] Gerlinger C, Schmelter T (2011) Determining the non-inferiority margin for patient reported outcomes. Pharm Stat 10(5):410–413 21932298 10.1002/pst.507

[52] Revicki D, Hays RD, Cella D, Sloan J (2008) Recommended methods for determining responsiveness and minimally important differences for patient-reported outcomes. J Clin Epidemiol 61(2):102–109 18177782 10.1016/j.jclinepi.2007.03.012

[53] Trigg A, Griffiths P (2021) Triangulation of multiple meaningful change thresholds for patient-reported outcome scores. Qual Life Res 30:2755–2764 34319532 10.1007/s11136-021-02957-4

[54] Turner RD, Birring SS (2023) Measuring cough: what really matters? J Thoracic Dis 15(4):2288 10.21037/jtd-23-230 PMC10183488 37197542

[55] Vertigan AE, Kapela SL, Birring SS, Gibson PG (2021) Feasibility and clinical utility of ambulatory cough monitoring in an outpatient clinical setting: a real-world retrospective evaluation. ERJ Open Res 7 (4). 10.1183/23120541.00319-2021 PMC8488350 34616839

[56] Birring S, Muccino D, Bacci ED, Vernon MK, Nguyen AM (2019) Defining minimal clinically important differences (MCID) on the Leicester Cough Questionnaire (LCQ): analyses of a phase 2 randomized controlled trial in chronic cough. J Allergy Clin Immunol 143 (2).

[57] Pornsuriyasak P, Thungtitigul P, Kawamatawong T, Birring SS, Pongmesa T (2017) Minimal clinically important differences (MCIDs) of the Thai version of the Leicester cough questionnaire for subacute and chronic cough. Value Health Regional Issues 12:57–62 10.1016/j.vhri.2017.03.009 28648317

[58] Eremenco S, Chen W-H, Blum SI, et al. (2022) Comparing patient global impression of severity and patient global impression of change to evaluate test–retest reliability of depression, non-small cell lung cancer, and asthma measures. Qual Life Res 31(12):3501–3512 35854060 10.1007/s11136-022-03180-5 PMC9587936

[59] Rhatigan K, Hirons B, Kesavan H, et al. (2023) Patient Global Impression of Severity Scale in Chronic Cough: validation and Formulation of Symptom Severity Categories. J Allergy Clin Immun Practice. 10.1016/j.jaip.2023.08.046 37678666

[60] Coon CD, Cook KF (2018) Moving from significance to real-world meaning: methods for interpreting change in clinical outcome assessment scores. Qual Life Res 27:33–40 28620874 10.1007/s11136-017-1616-3

[61] Domingo C, Quirce S, Dávila I, et al. (2024) Cough severity visual analog scale scores and quality of life in patients with refractory or unexplained chronic cough. Respiratory Investigation 62(6):987–994 39197381 10.1016/j.resinv.2024.08.005

[62] Cella D, Hahn EA, Dineen K (2002) Meaningful change in cancer-specific quality of life scores: differences between improvement and worsening. Qual Life Res 11:207–221 12074259 10.1023/a:1015276414526

