# Supplementary material for: Psychometric validation of the severity of chronic cough diary, leicester cough questionnaire, and a cough severity visual analogue scale in patients with refractory chronic cough
Source: J Patient Rep Outcomes. 2025 Jun 11;9:65. doi: 10.1186/s41687-025-00888-z (PMC12158865; doi:10.1186/s41687-025-00888-z)
Supplement: Supplementary file 1 — Supplementary Material 1 [file 41687_2025_888_MOESM1_ESM.docx]

**SUPPLEMENTARY MATERIALS I: Additional responsiveness analyses**

Table A. Responsiveness according to changes in PGI-C anchor ratings between BL and WK12

| **PRO Score (range)**  PGI-S Anchor Groups | **n** | **Mean Change Score (SD)** | **Median Change Score**  **(Min-Max)** | **Within groups effect size** | **Between Groups p-value** |
| --- | --- | --- | --- | --- | --- |
| **SCCD Item 1 - Cough Frequency (0-4)** |  |  |  |  |  |
| Improved: A little better / Much better / Very much better | 188 | -0.92 (0.79) | -0.93 (-3.0, 2.1) | -1.70 | <.001 |
| Stable: No change | 59 | -0.19 (0.64) | -0.26 (-1.0, 2.8) | -0.32 |  |
| Worsened: A little worse / Much worse / Very much worse | 8 | -0.03 (0.57) | 0.01 (-0.8, 0.8) | -0.04 |  |
| **SCCD Item 5 – Cough Severity (0-4)** |  |  |  |  |  |
| Improved: A little better / Much better / Very much better | 188 | -0.85 (0.75) | -0.86 (-3.0, 3.1) | -1.52 | <.001 |
| Stable: No change | 59 | -0.16 (0.62) | -0.14 (-1.2, 2.6) | -0.25 |  |
| Worsened: A little worse / Much worse / Very much worse | 8 | -0.15 (0.67) | -0.13 (-1.3, 0.6) | -0.20 |  |
| **LCQ Total (3-21)** |  |  |  |  |  |
| Improved: A little better / Much better / Very much better | 199 | 3.54 (3.32) | 3.14 (-4.6, 14.3) | 1.33 | <.001 |
| Stable: No change | 61 | 0.30 (2.04) | 0.45 (-7.1, 4.1) | 0.09 |  |
| Worsened: A little worse / Much worse / Very much worse | 8 | -0.38 (1.99) | -0.64 (-3.8, 2.9) | -0.09 |  |
| **LCQ Physical domain (1-7)** |  |  |  |  |  |
| Improved: A little better / Much better / Very much better | 199 | 0.98 (0.98) | 0.88 (-1.3, 3.9) | 1.13 | <.001 |
| Stable: No change | 61 | 0.10 (0.73) | 0.25 (-2.1, 2.4) | 0.10 |  |
| Worsened: A little worse / Much worse / Very much worse | 8 | -0.22 (0.53) | -0.31 (-0.8, 0.6) | -0.20 |  |
| **Cough Severity VAS (0-100)** |  |  |  |  |  |
| Improved: A little better / Much better / Very much better | 185 | -29.05 (23.42) | -25.24 (-92.4, 35.0) | -1.78 | <.001 |
| Stable: No change | 59 | -4.05 (12.09) | -2.14 (-55.0, 10.6) | -0.22 |  |
| Worsened: A little worse / Much worse / Very much worse | 8 | -0.07 (13.58) | -0.05 (-17.6, 24.3) | -0.00 |  |

Within groups effect size: mean change score divided by the SD of the score at Baseline. Between groups p-value: F-test from a one-way ANOVA

Table B. Responsiveness according to changes in Awake Cough Count ratings between BL and WK12

| **PRO Score (range)**  PGI-S Anchor Groups | **n** | **Mean Change Score (SD)** | **Median Change Score**  **(Min-Max)** | **Within groups effect size** | **Between Groups p-value** |
| --- | --- | --- | --- | --- | --- |
| **SCCD Item 1 – Cough Frequency (0-4)** |  |  |  |  |  |
| Improved: change score <= -30% | 139 | -1.01 (0.82) | -1.00 (-3.0, 1.4) | -1.94 | <.001 |
| Stable: -30% < change score < 30% | 82 | -0.43 (0.54) | -0.30 (-2.0, 0.6) | -0.97 |  |
| Worsened: change score >= 30% | 26 | -0.03 (0.91) | -0.27 (-1.1, 2.8) | -0.04 |  |
| **SCCD Item 5 - Cough Severity (0-4)** |  |  |  |  |  |
| Improved: change score <= -30% | 139 | -0.94 (0.69) | -0.86 (-3.0, 0.5) | -1.75 | <.001 |
| Stable: -30% < change score < 30% | 82 | -0.41 (0.61) | -0.43 (-1.7, 1.0) | -0.81 |  |
| Worsened: change score >= 30% | 26 | 0.03 (0.96) | -0.07 (-1.3, 3.1) | 0.05 |  |
| **LCQ Total (3-21)** |  |  |  |  |  |
| Improved: change score <= -30% | 145 | 3.88 (3.37) | 3.63 (-4.9, 14.3) | 1.37 | <.001 |
| Stable: -30% < change score < 30% | 85 | 1.20 (2.25) | 0.73 (-5.2, 8.0) | 0.44 |  |
| Worsened: change score >= 30% | 27 | 0.15 (2.55) | -0.04 (-7.1, 4.6) | 0.05 |  |
| **LCQ Physical domain (1-7)** |  |  |  |  |  |
| Improved: change score <= -30% | 145 | 1.09 (1.00) | 1.00 (-1.8, 3.9) | 1.18 | <.001 |
| Stable: -30% < change score < 30% | 85 | 0.33 (0.66) | 0.38 (-1.4, 2.0) | 0.36 |  |
| Worsened: change score >= 30% | 27 | -0.04 (0.83) | 0.00 (-2.1, 2.0) | -0.04 |  |
| **Cough Severity VAS (0-100)** |  |  |  |  |  |
| Improved: change score <= -30% | 136 | -30.11 (24.81) | -27.64 (-92.4, 13.1) | -1.77 | <.001 |
| Stable: -30% < change score < 30% | 83 | -13.19 (16.20) | -8.14 (-68.6, 10.6) | -0.94 |  |
| Worsened: change score >= 30% | 25 | -5.92 (17.79) | -5.36 (-47.1, 35.0) | -0.27 |  |

Within groups effect size: mean change score divided by the SD of the score at Baseline. Between groups p-value: F-test from a one-way ANOVA

**SUPPLEMENTARY MATERIALS II: Psychometric validation results for Cough Severity VAS**

***Quality of completion and descriptive statistics:*** Participant completion over a 7-day period (up to and including the respective study visit) at baseline, WK4, and WK12 was relatively high for the Cough Severity VAS (87.0% - 88.1% missing no days or a single day). For the Cough Severity VAS, responses of 0-9 were considered ceiling effects and responses of 91-100 were considered floor effects. No floor and ceiling effects were observed at baseline for the Cough Severity VAS.

***Reliability:*** Test-retest reliability was good to excellent for the Cough Severity VAS (ICC ≥0.935) among participants that were defined as stable between WK3 and WK4 according to PGI-S ratings and Awake Cough Count readings (Table 3).

***Known-groups comparisons:*** Anticipated linear trends in group means were observed for Cough Severity VAS scores among groups categorized according to PGI-S ratings and objective Awake Cough Counts (Figure 2). For all scores, differences between groups defined according to PGI-S ratings were large (effects sizes 1.31 – 2.86) in accordance with pre-specified hypotheses. Smaller differences between groups defined according to Awake Cough Count were observed as expected (small to large effect sizes; 0.44 – 0.85). All differences between groups were statistically significant (p<0.001).

***Responsiveness:*** Statistically significant differences (p<0.001) in Cough Severity VAS mean change scores were observed between ‘improved’, ‘stable’ and ‘worsened’ groups defined according to all external measures. Ability to detect improvement was supported with the greatest mean change scores and large effect sizes observed for Cough Severity VAS scores among participants classified as ‘improved’ according to PGI-S ratings (Table 5). Similar results were observed when change was defined according to PGI-C ratings and Awake Cough Count (Supplementary Materials I)

***Interpretation of scores:*** Correlations between changes in Cough Severity VAS scores with selected anchors (PGI-S, PGI-C, and Awake Cough Count) between BL and WK12 were all acceptable (i.e., r≥0.3). Estimates of MWPC for Cough Severity VAS scores, based on interpretation of ROC curves and discriminant analysis are outlined in Table 6. AUC values for Cough Severity VAS were 0.785 (PGI-S), 0.845 (PGI-C) and 0.705 (Awake Cough Count). The triangulated threshold for the Cough Severity VAS score was -17.73 (95% CI [-22.95, -12.51]).
